# Facile preparation of highly luminescent CdTe quantum dots within hyperbranched poly(amidoamine)s and their application in bio-imaging

**DOI:** 10.1186/1556-276X-9-115

**Published:** 2014-03-13

**Authors:** Yunfeng Shi, Lin Liu, Huan Pang, Hongli Zhou, Guanqing Zhang, Yangyan Ou, Xiaoyin Zhang, Jimin Du, Wangchuan Xiao

**Affiliations:** 1 School of Chemistry and Chemical Engineering, Anyang Normal University, Anyang 455000, People’s Republic of China; 2 School of Resources and Chemical Engineering, Sanming University, Sanming 365004, People’s Republic of China

**Keywords:** Hyperbranched poly(amidoamine)s, CdTe quantum dots, Fluorescence, Nanocomposites, Bio-imaging

## Abstract

A new strategy for facile preparation of highly luminescent CdTe quantum dots (QDs) within amine-terminated hyperbranched poly(amidoamine)s (HPAMAM) was proposed in this paper. CdTe precursors were first prepared by adding NaHTe to aqueous Cd^2+^ chelated by 3-mercaptopropionic sodium (MPA-Na), and then HPAMAM was introduced to stabilize the CdTe precursors. After microwave irradiation, highly fluorescent and stable CdTe QDs stabilized by MPA-Na and HPAMAM were obtained. The CdTe QDs showed a high quantum yield (QY) up to 58%. By preparing CdTe QDs within HPAMAM, the biocompatibility properties of HPAMAM and the optical, electrical properties of CdTe QDs can be combined, endowing the CdTe QDs with biocompatibility. The resulting CdTe QDs can be directly used in biomedical fields, and their potential application in bio-imaging was investigated.

## Background

Quantum dots (QDs), also referred to as semiconductor nanocrystals, exhibit unique size and shape-dependent optical and electronic properties [1-7]. In recent years, significant progress has been made from the synthesis of QDs to the fabrication of nanodevices and nanostructured materials [8-14].

Until now, various ways have been developed to synthesize high-quality QDs, such as the nonaqueous trioctylphosphine oxide (TOPO)/trioctylphosphine (TOP) technique [15-18], the aqueous route with small thiols [2,3,19-23] or dendritic polymers [24-28] as stabilizers, and the biometric template method [29]. The fluorescent QDs stabilized by small thiols or TOPO are inherently instable and should be stabilized by matrix materials in order to realize their successful applications, while the QDs prepared with dendritic polymers as stabilizers and nanoreactors can be directly applied to many fields. The dendritic polymers have three-dimensional globular architecture, numerous cavities, and plenty of peripheral functional groups, which offer dendritic polymers the capability of *in situ* preparing QDs with controlled size. The QDs prepared within dendritic polymer integrate the optical, electrical properties of QDs and the biocompatibility properties of polymers together, and they are easy to form films or to assemble on substrates. However, the low-quantum yield (QY) and the broad emission spectrum of QDs prepared within dendritic polymers still need to be improved further.

Now, preparation of CdS QDs within dendritic polymers has been reported [23-28]. However, the low QY and the broad emission spectrum of CdS QDs are still unresolved. There is also one work relating to preparing CdTe QDs within poly(amidoamine)s dendrimers (PAMAM) [30]; however, the highly fluorescence of CdTe QDs prepared within dendritic polymers has not been resolved. In our experiment, we found that if CdTe QDs were directly prepared within dendritic PAMAM without other stabilizers, they were easy to be oxidized and had very weak fluorescence even if microwave heating was used. There are also some works relating on modification of preformed CdTe QDs by dendritic PAMAM [31,32]. By forming covalent bonds between CdTe QDs and PAMAM, CdTe/PAMAM nanocomposites were prepared. Nevertheless, this method is used for the functionalization of preformed CdTe QDs but not for *in situ* preparation of CdTe QDs, and the CdTe/PAMAM nanocomposites might form large aggregation or self-assembly. Compared with this route, *in situ* preparation of fluorescent CdTe QDs within dendritic polymers would be more convenient and effective.

In this paper, we propose a new method to synthesize highly fluorescent and stable CdTe QDs within hyperbranched poly(amidoamine)s (HPAMAM). HPAMAM was introduced to coat the CdTe precursors (not CdTe QDs) stabilized by mercaptopropionic sodium (MPA-Na). By this way, the growth of CdTe QDs can be further controlled, and the CdTe QDs can be endowed with biocompatibility by HPAMAM. After microwave irradiation, highly fluorescent and stable CdTe QDs stabilized by MPA-Na and HPAMAM were obtained. The resulting CdTe/HPAMAM nanocomposites combine the optical, electrical properties of CdTe QDs and the biocompatibility properties of HPAMAM together. They can be directly used in biomedical fields, and their potential application in bio-imaging was investigated.

## Methods

HPAMAM with amine terminals was synthesized according to our previous work [14]. After endcapping by palmitoyl chloride, the weight average molecular weight (Mw) of HPAMAM measured by gel permeation chromatography (GPC) was about 1.1 × 10^4^ and the molecular weight polydispersity (PDI) was 2.7. CdCl_2_ · 2.5H_2_O (99%); NaBH_4_ (96%), tellurium powder (99.999%), and methanol were purchased from Sinopharm Chemical Reagent Co., Ltd., Shanghai, China. 3-Mercaptopropionic acid (MPA, >99%) was purchased from Fluka, St. Louis, MO, USA. The ultrapure water with 18.2 MΩ · cm was used in all experiments.

MPA (0.26 mL) was added to 100 mL CdCl_2_ (1.25 mmol) aqueous solution. After stirring for several hours, the aqueous solution was diluted to 950 mL, followed by adjusting the pH value to 8 with 1 M NaOH. After deaeration with N_2_ for 30 min, 50 mL oxygen-free NaHTe solution was injected at 5°C under vigorous stirring, thus CdTe precursor solution was obtained.

Proper amounts of HPAMAM (for example, 120 mg) was dissolved in 2 mL H_2_O in a one-neck flask, and then, 100 mL CdTe precursor solution was added. The mixture was deaerated with N_2_ for 15 min, followed by stirring for 24 h. Then, the mixture was irradiated at different times under ordinary pressure microwave (SINEO Shanghai Xinyi, Shanghai, China, 200 W, 100°C) to get a series of samples with various colors. The final CdTe/HPAMAM nanocomposites were abbreviated as CdTe/HPAMAM120. CdTe QDs prepared within 40, 80, and 200 mg HPAMAM were called CdTe/HPAMAM40, CdTe/HPAMAM80, and CdTe/HPAMAM200, respectively. The CdTe QDs stabilized by MPA-Na without HPAMAM were called CdTe/MPA-Na.

Cell imaging was characterized by confocal laser scanning microscopy (CLSM). HeLa cells (1 × 10^5^ cells per well) were seeded on coverslips in 12-well tissue culture plates. After incubating the cells for 24 h, the CdTe/HPAMAM120 nanocomposites (obtained on heating for 80 min) in 200 μL Dulbecco's modified Eagle's medium (DMEM) were added into the wells and the cells were incubated at 37°C for 6 h. After washed with PBS, the cells were fixed with 4% formaldehyde for 30 min at room temperature. Then, the slides were rinsed with PBS for two times. The slides were mounted and observed by a LSM 510META.

No postpreparative treatment was performed on any as-prepared samples for optical characterization. pH values were measured by a Starter 3C digital pH meter, Ohaus, Parsippany, New Jersey, USA. Transmission electron microscopy (TEM), selected area electron diffraction (SAED), and elemental characterization were done on a JEOL 2010 microscope, Akishima-shi, Japan, with energy-dispersive X-ray spectrometer (EDS) at an accelerating voltage of 200 kV. X-ray powder diffraction (XRD) spectrum was taken on D/max-2550/PC X-ray diffractometer operated at 40 kV voltage and 40 mA current with Cu Ka radiation. For the XRD measurement, the CdTe QDs were rotary evaporated to remove water and then dried under vacuum. UV-visible (vis) spectra were recorded on a Varian Cary 50 UV/Vis spectrometer, Agilent Technologies, Inc., Santa Clara, CA, USA. Emission spectra were collected using a Varian Cary spectrometer. Dynamic light scattering (DLS) measurements were performed in aqueous solution at 25°C by using Zetasizer Nano S (Malvern Instruments Ltd., Malvern, Worcestershire, UK). The infrared measurements were performed on a Varian 800 Fourier transform infrared spectroscopy (FTIR) spectrometer. Thermogravimetric analysis (TGA) was performed under nitrogen on a STA 409 PC thermal analyzer, Netzsch, Germany.

The QY of CdTe QDs was measured according to the methods described in [33] using rhodamine 6G as a reference standard (QY = 95%). The absorbance for the standard and the CdTe samples at the absorption peak and the fluorescence spectra of the same solutions excited at wavelengths of absorption peaks were measured, respectively. The area of the fluorescence spectrum from the fully corrected fluorescence spectrum was calculated. Rhodamine 6G (ethanol) and the CdTe solutions (0.03 < the absorbance at the excitation wavelength < 0.1) were used in the measurements. The QY was calculated according to the following equation:

(1) $$\varphi_{x}=\varphi_{s}\left(\frac{F_{x}}{F_{s}}\right)\;\left(\frac{A_{s}}{A_{x}}\right)\;{\left(\frac{\eta_{x}}{\eta_{s}}\right)}^{2}$$

where the subscripts s and x denote standard (rhodamine 6G) and test samples, respectively, *φ* is QY, *F* is the area of integrated fluorescence intensity, *A* is the absorbance at the absorption peak, and *η* is the refractive index of the solvent.

## Results and discussion

HPAMAM have three-dimensional topological structures, many inner cavities, and a large amount of terminal functional groups. They have low cytotoxicity and have been widely used in biomedical science, such as gene transfections and drug delivery [34-36]. Based on this, we proposed new preparation strategies that combine the biomedical properties of dendritic polymers with the synthesis of CdTe QDs together in this paper. HPAMAM was introduced to stabilize the CdTe precursors (not CdTe QDs). After microwave irradiation, highly fluorescent and stable CdTe QDs stabilized by MPA-Na and HPAMAM were obtained. By preparing CdTe QDs within HPAMAM, the biocompatibility properties of HPAMAM and the optical, electrical properties of CdTe QDs can be combined, endowing the CdTe QDs with biocompatibility.

**Figure 1:**
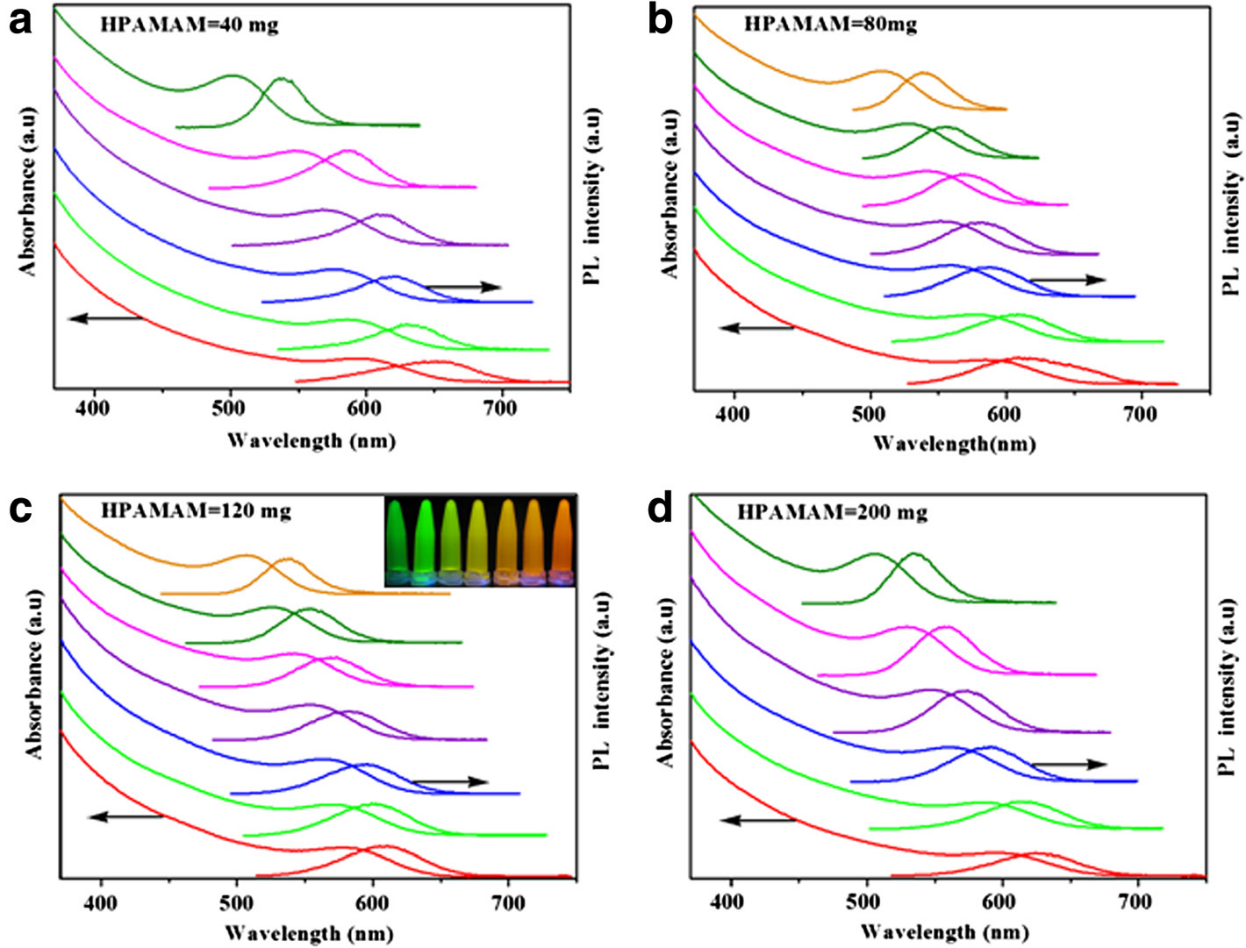
**UV-vis absorption and PL spectra of CdTe QDs.** UV-vis absorption and PL spectra **(a-d)** of *in situ* prepared CdTe QDs obtained by adding different amounts of HPAMAM and heating at various times. The inset in **(c)** shows fluorescent photographs of as-prepared CdTe QDs under UV irradiation. Photoluminescence spectra were recorded with excitation at 370 nm.

Figure 1 gives the typical evolutions of both absorption and photoluminescence spectra of CdTe QDs prepared within HPAMAM. The CdTe QDs were obtained by irradiation every 40 min under ordinary pressure microwave. All the UV-vis spectra show well-resolved absorption peaks, and the absorption peak shifted to a longer wavelength along with the heating time, indicating the growth of CdTe QDs. The maximum peak of PL emission also shows red shift. The fluorescence color of CdTe QDs under UV light changed from green to yellow and orange with prolonging refluxed time (inset in Figure 1c). The size of CdTe QDs can be estimated from the absorption peaks using Peng's empirical formula [37]. From the absorption peaks, the Peng's empirical formula predicts that the diameter of CdTe QDs is from 2.5 to 3.5 nm. The absorption and photoluminescence spectra of CdTe QDs stabilized only by MPA-Na can be seen in Additional file 1: Figure S1 in the supporting information.

**Figure 2:**
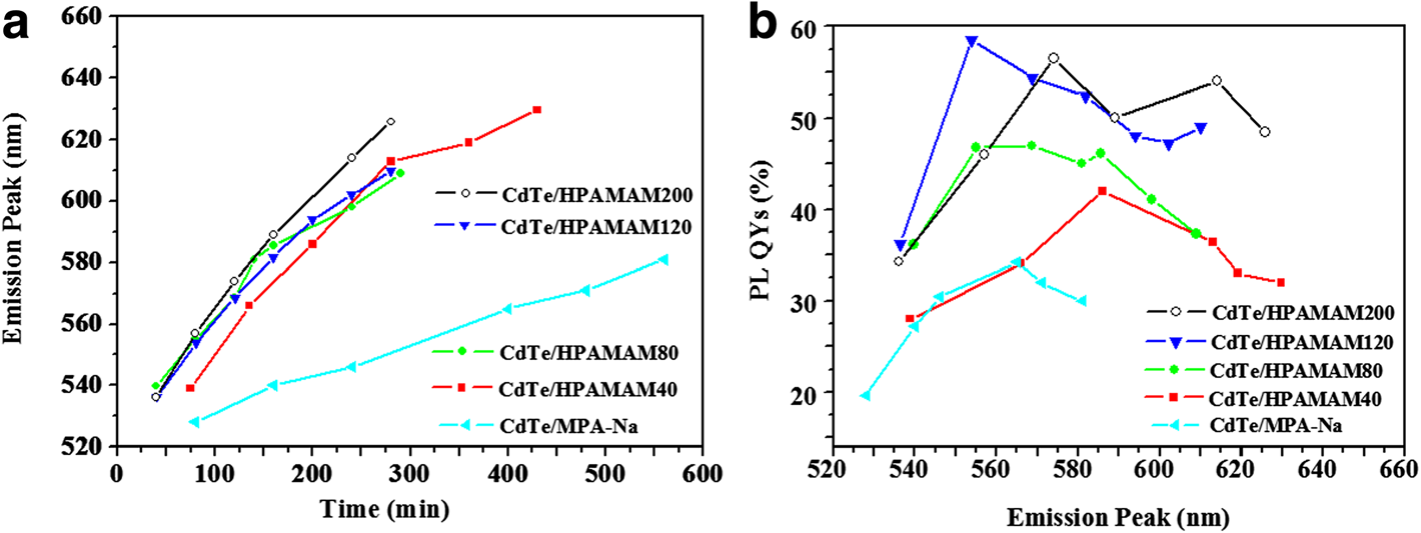
**CdTe PL peak position vs reaction time (a) and PL QYs vs PL wavelength (b).** The reaction temperature was 100°C, and the amount weight of HPAMAM used for synthesizing CdTe QDs were 40, 80, 120, and 200 mg, respectively. The CdTe QDs synthesized without HPAMAM were called CdTe/MPA-Na.

As shown in Figure 2a, the CdTe QDs prepared within different amounts of HPAMAM (40, 80, 120, and 200 mg) has a similar growth process, while the growth rate of CdTe QDs prepared with MPA-Na as stabilizers was much slower. We speculate that when HPAMAM macromolecules are added, a great amount of amines exist and part of MPA-Na might be replaced by the amines of HPAMAM. Because the surface of CdTe QDs coated by HPAMAM is not as compact as that of CdTe QDs coated by small MPA-Na molecules, the growth rate of CdTe QDs coated with HPAMAM has a more quick growth rate. As shown in Figure 2b, the CdTe QDs prepared within different amounts of HPAMAM exhibited parabola-like curves of the emission wavelength and the QYs of QDs, which were similar with the general phenomena during the growth of MPA-Na capped CdTe QDs (CdTe/MPA-Na) [38-40]. From Figure 2b, we also can see that the QY of CdTe QDs is positively correlated with the content of HPAMAM added in general trend. When more HPAMAM macromolecules were added, the electrostatic interactions between the amines of HPAMAM and MPA-Na were enhanced. The HPAMAM shell acted as surface passivation ligands to get rid of surface traps caused by dangling bonds, resulting in the high QY of CdTe QDs.

The QY of CdTe/MPA-Na in maximum is 33%, while the best aliquot with emission maximum at 554 nm of CdTe/HPAMAM120 show a high QY up to 58%, which is almost two times of that of CdTe/MPA-Na. Consequently, we can see that HPAMAM plays very important roles in enhancing the QY of CdTe QDs. The corresponding UV-vis spectrum and PL spectrum of CdTe/HPAMAM120 with emission maximum at 554 nm can be seen in the second set of curves from top to bottom in Figure 1c, and the corresponding fluorescent photograph under UV irradiation is located in the second place in the inserted photograph of Figure 1c.

**Figure 3:**
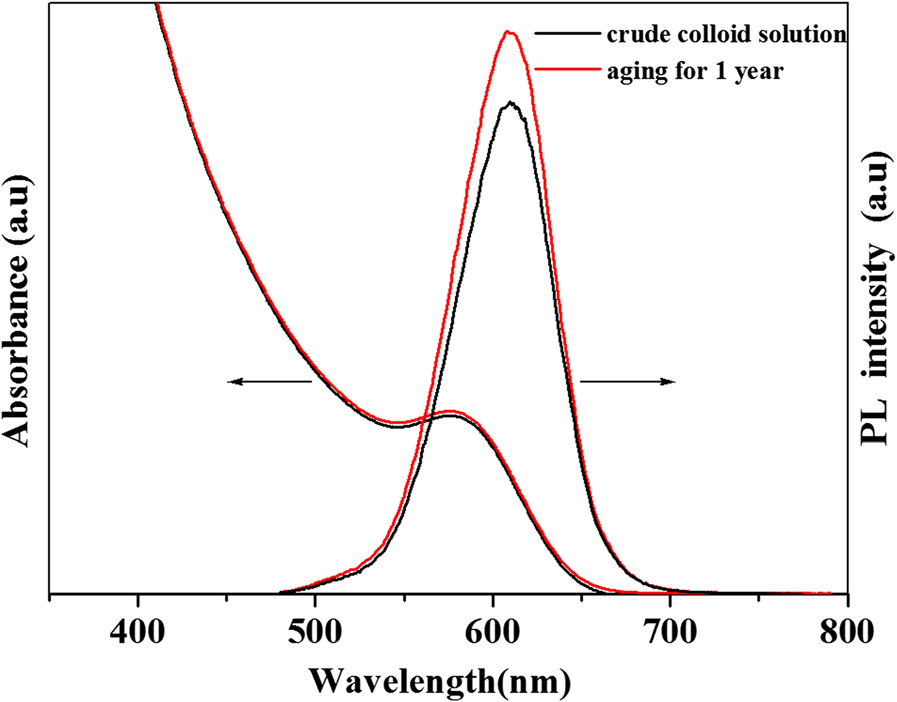
**The absorption and emission spectra of CdTe aqueous solution.** Prepared within 120 mg HPAMAM before and after being aged for 1 year. The CdTe QDs sample was synthesized by heating for 280 min, and its absorption peak was 577 nm.

The stability of these highly luminescent CdTe QDs prepared within HPAMAM was investigated. After synthesis, samples taken at different reflux times were used to investigate their aqueous stability, and we found that they all showed good stability in the aqueous phase even for 1 year. No obvious precipitations were observed for these samples. Figure 3 shows the emission and absorption spectra of CdTe QDs synthesized within 120 mg HPAMAM before and after being kept for 1 year. After being kept for 1 year, the absorption of CdTe QDs (the excitonic absorption peak at 577 nm) had a slight change and the luminescence intensity increased a little. We suppose that the surface defect might be eliminated after aging, so this causes a slight increasement of luminescence intensity.

**Figure 4:**
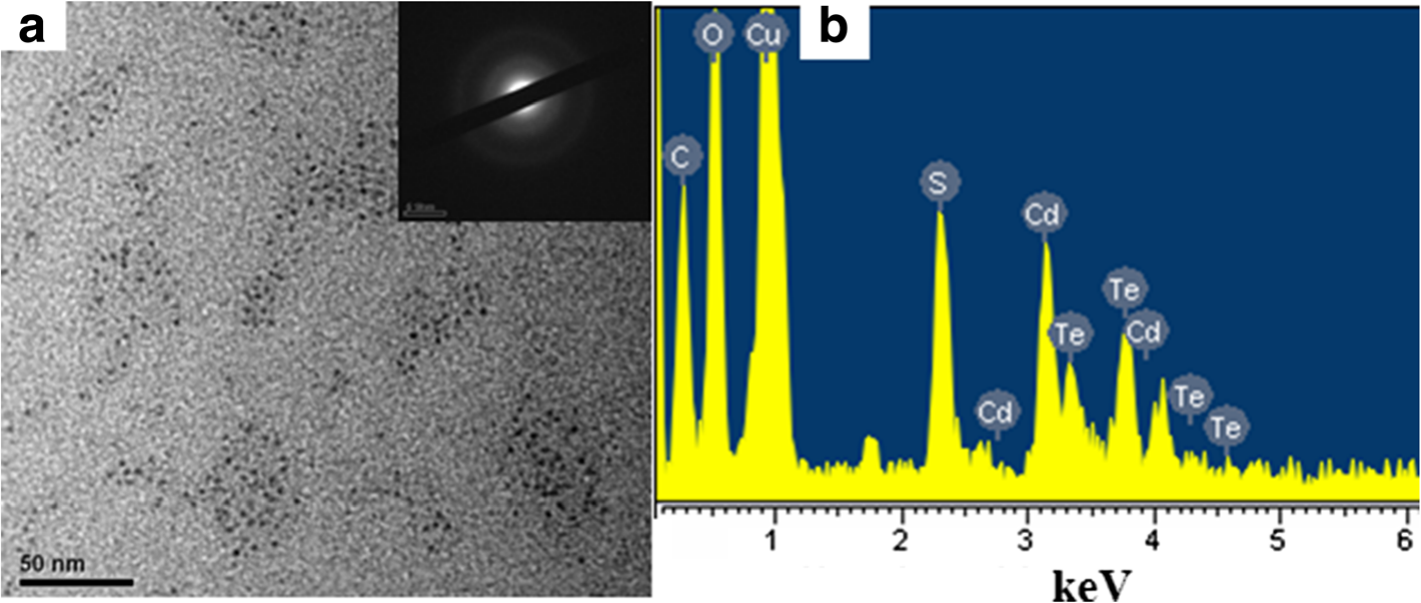
**TEM image (a) and (b) EDS spectrum of CdTe/HPAMAM120.** The excitonic absorption peak at 577 nm. Inset in **(a)**, the corresponding SAED pattern.

Figure 4a presents the TEM image of CdTe/HPAMAM120 (the excitonic absorption peak at 577 nm). The particle size distribution is quite uniform, and the average diameter of CdTe QDs is about 3.4 nm. The SAED pattern inserted in Figure 4a shows that the synthesized fluorescent nanoparticles are polycrystalline. The corresponding EDS spectrum (Figure 4b) gives the signals of Cd and Te elements, confirming the existence of CdTe QDs.

**Figure 5:**
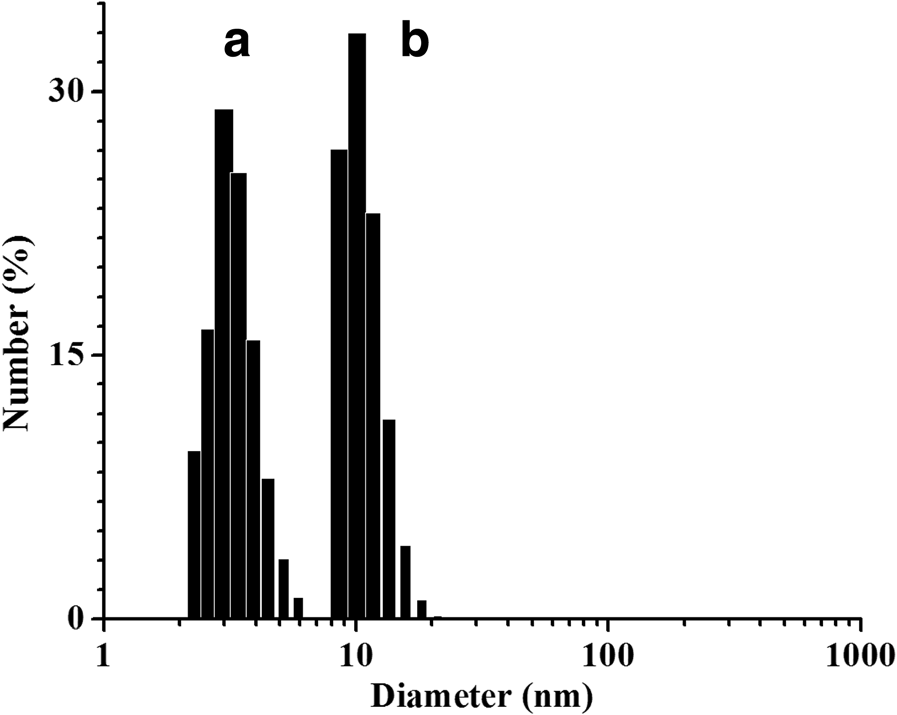
Size distribution of HPAMAM (a) and CdTe/HPAMAM120 (the excitonic absorption peak at 577 nm) nanocomposites (b), measured by DLS.

The hydrodynamic diameters of CdTe/HPAMAM120 nanocomposites and pure HPAMAM were measured by dynamic light scattering technique. Figure 5 gives the particle size distributions of pure HPAMAM and CdTe/HPAMAM120 (the excitonic absorption peak at 577 nm). It can be found that the average diameter of HPAMAM was 2.9 nm, while the CdTe/HPAMAM120 nanocomposites had an average diameter of 10.1 nm. Considering the size of HPAMAM, there is an increase of the diameter by 7.2 nm, indicating the formation of CdTe/HPAMAM assemblies. That is, the CdTe QDs were synthesized within several HPAMAM macromolecules.

**Figure 6:**
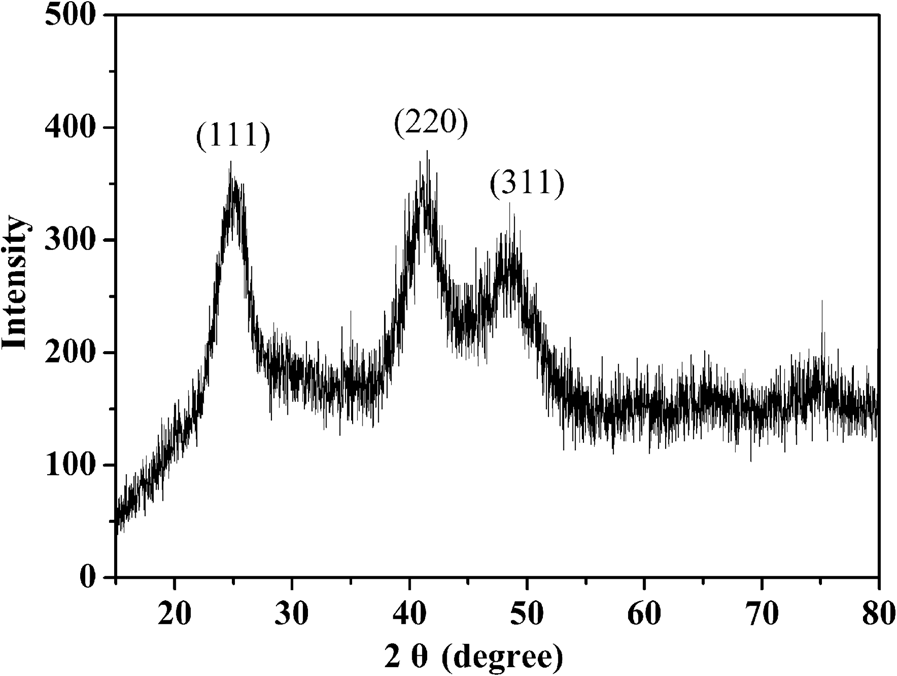
XRD spectrum of CdTe/HPAMAM120 (the excitonic absorption peak at 577 nm).

Figure 6 shows XRD pattern of CdTe/HPAMAM120 (the excitonic absorption peak at 577 nm). The CdTe QDs exhibit X-ray diffraction pattern consistent with cubic (zinc blende) CdTe, as represented by the broad diffraction peaks at 24.8° (111), 41.5° (220), and 48.6° (311).

**Figure 7:**
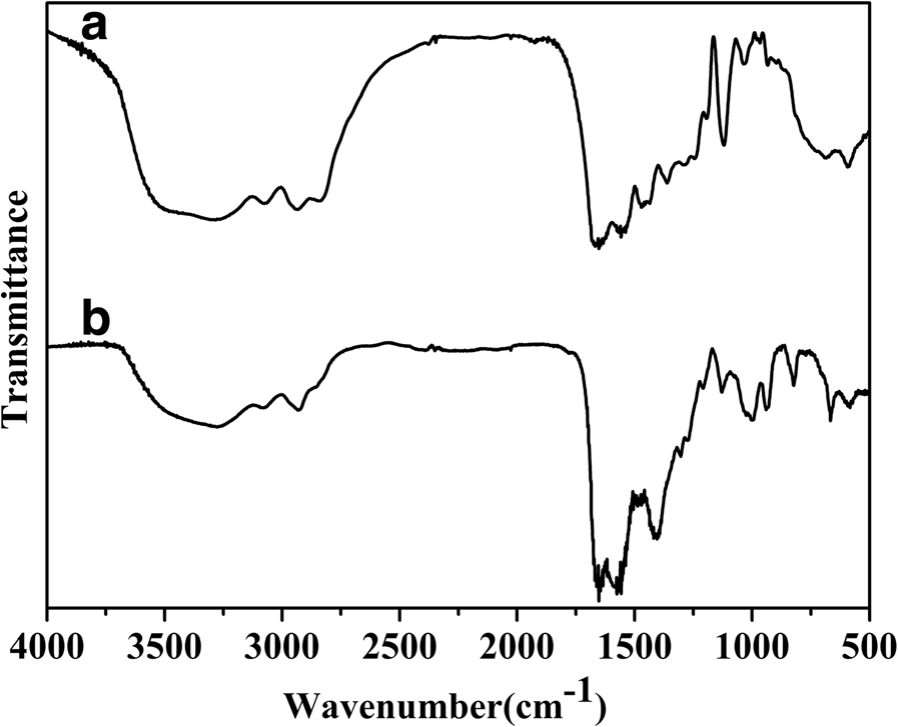
FTIR spectra of HPAMAM (curve a) and CdTe/HPAMAM120 (the excitonic absorption peak at 577 nm) (curve b).

FTIR spectra of CdTe/HPAMAM120 and pure HPAMAM are shown in Figure 7. The bands at 2,929 and 2,846 cm^-1^ in both curves correspond to asymmetric - CH_2_- stretching vibration and symmetric - CH_2_- stretching vibration, respectively. The characteristic bands assigned to amides I and II for HPAMAM are at 1,661 and 1,557 cm^-1^, respectively, while the band positions of amides I and II slightly shift to 1,652 and 1,575 cm^-1^ for the CdTe/HPAMAM120 nanocomposites. These frequency shifts in FTIR can be attributed to the coordination interactions between CdTe QDs and HPAMAM through their amine groups.

**Figure 8:**
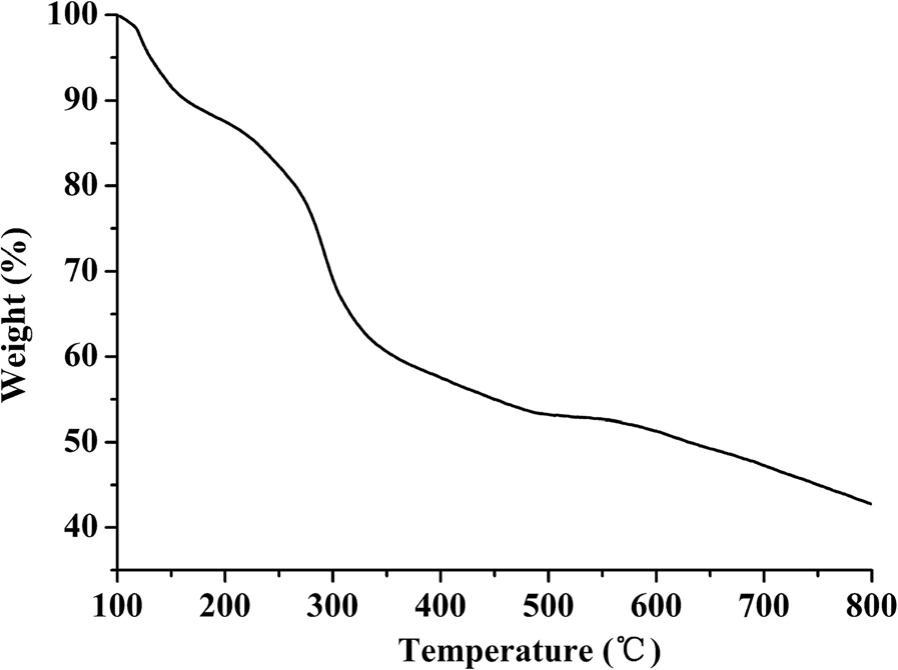
**TGA weight loss curves of CdTe/HPAMAM120 (the excitonic absorption peak at 577 nm).** The heating rate was 20°C/min.

The composition of CdTe/HPAMAM120 was measured by TGA, and the results are shown in Figure 8. The weight loss below 200°C is due to the removal of absorbed physical and chemical water. TGA thermogram shows a great weight loss in the temperature range 200°C to 500°C, which is the range of decomposition temperature for HPAMAM. At 800°C, the weight is 42 wt.%, corresponding to the content of CdTe QDs in the CdTe/HPAMAM120 nanocomposites.

**Figure 9:**
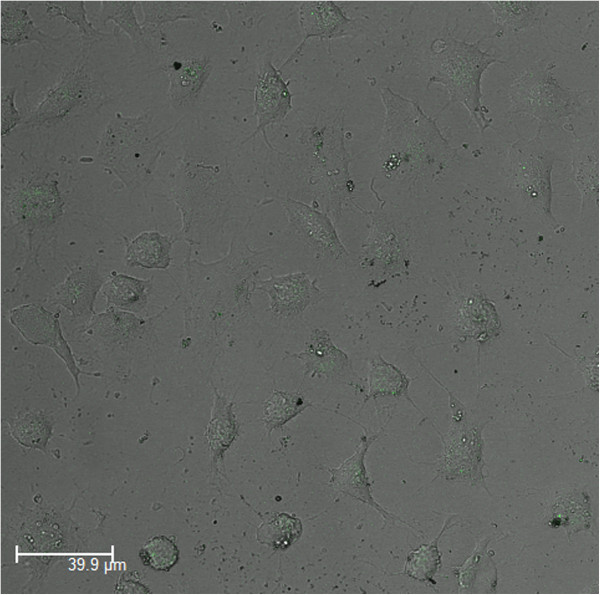
Confocal micrograph of HeLa macrophage incubated with CdTe/HPAMAM120 (the excitonic absorption peak at 527 nm).

To investigate the potential application of the CdTe/HPAMAM nanocomposites in bio-imaging, the CdTe/HPAMAM120 nanocomposites (the excitonic absorption peak at 527 nm) were used for HeLa cell imaging evaluated by CLSM. HPAMAM-capped CdTe QDs were seen to be internalized into HeLa cells, as shown in Figure 9. As the hyperbranched polyamines could be endocytosed by the cells [41,42], the CdTe QDs capped by HPAMAM also can be internalized into cells without other transfection reagent. HPAMAM has been widely used in biomedical science, such as gene transfections and drug delivery, so the resulting high QY CdTe/HPAMAM nanocomposites might be well applied in gene transfection and drug delivery which can be monitored by their own fluorescent CdTe QDs.

## Conclusions

In conclusion, a new strategy for preparing highly luminescent CdTe QDs within amine-terminated HPAMAM was proposed in this paper. The resulting CdTe/HPAMAM nanocomposites showed a high QY up to 58%. They combined the optical, electrical properties of CdTe QDs and the biocompatibility property of HPAMAM together. They could be directly used in biomedical fields, and their potential application in bioimaging was also investigated. Potential applications in gene transfection and drug delivery may be ideal because the fluorescent CdTe QDs can be used to monitor the entire process.

## Competing interests

The authors declare that they have no competing interests.

## Authors' contributions

YS, LL, and HP carried out all the experiments and drafted the manuscript. HZ, GZ, YO, XZ, WX, and JD participated in preparing and characterizing quantum dots. All authors read and approved the final manuscript.

## Supplementary Material

Additional file 1 The absorption and photoluminescence spectra of CdTe QDs stabilized only by MPA-Na. Click here for file
