# Supplementary material for: Facile preparation of highly luminescent CdTe quantum dots within hyperbranched poly(amidoamine)s and their application in bio-imaging
Source: Nanoscale Res Lett. 2014 Mar 13;9(1):115. doi: 10.1186/1556-276X-9-115 (PMC4007777; doi:10.1186/1556-276X-9-115)
Supplement: Additional file 1 — The absorption and photoluminescence spectra of CdTe QDs stabilized only by MPA-Na. [file 1556-276X-9-115-S1.doc]

Supplementary Material:

Facile preparation of highly luminescent CdTe quantum dots within hyperbranched poly(amidoamine)s and their application in bio-imaging

Yunfeng shi,* Lin Liu*, Huan Pang*, Hongli Zhou, Guanqing Zhang, Yangyan Ou, Xiaoyin Zhang and Jimin Du

School of Chemistry and Chemical Engineering, Anyang Normal University, Anyang 455000, People's Republic of China.

*E-mail: shiyunfeng2009@gmail.com (Y. Shi), liulin82414@yahoo.com.cn (L. Liu), huanpangchem@hotmail.com (H. Pang).

Phone: +372-2900-040. Fax: +372-2900-040.

**Fig. S1.** The absorption and photoluminescence spectra of CdTe QDs stabilized only by MPA-Na.
